# Survival and Pulmonary Injury After Neonatal Sepsis: PD1/PDL1's Contributions to Mouse and Human Immunopathology

**DOI:** 10.3389/fimmu.2021.634529

**Published:** 2021-03-03

**Authors:** Eleanor A. Fallon, Chun-Shiang Chung, Daithi S. Heffernan, Yaping Chen, Monique E. De Paepe, Alfred Ayala

**Affiliations:** ^1^Division of Surgical Research, Department of Surgery, Alpert Medical School of Brown University, Rhode Island Hospital, Providence, RI, United States; ^2^Department of Surgery, Providence Veterans Affairs Medical Center, Providence, RI, United States; ^3^Department of Pathology, Women & Infants Hospital and Alpert Medical School of Brown University, Providence, RI, United States

**Keywords:** programmed cell death receptor, sepsis, lung injury, immunopathology, innate immunity, neonatal, survival, endothelial cell culture

## Abstract

Morbidity and mortality associated with neonatal sepsis remains a healthcare crisis. PD1^−/−^ neonatal mice endured experimental sepsis, in the form of cecal slurry (CS), and showed improved rates of survival compared to wildtype (WT) counterparts. End-organ injury, particularly of the lung, contributes to the devastation set forth by neonatal sepsis. PDL1^−/−^ neonatal mice, in contrast to PD1^−/−^ neonatal mice did not have a significant improvement in survival after CS. Because of this, we focused subsequent studies on the impact of PD1 gene deficiency on lung injury. Here, we observed that at 24 h post-CS (but not at 4 or 12 h) there was a marked increase in pulmonary edema (PE), neutrophil influx, myeloperoxidase (MPO) levels, and cytokine expression sham (Sh) WT mice. Regarding pulmonary endothelial cell (EC) adhesion molecule expression, we observed that Zona occludens-1 (ZO-1) within the cell shifted from a membranous location to a peri-nuclear location after CS in WT murine cultured ECs at 24hrs, but remained membranous among PD1^−/−^ lungs. To expand the scope of this inquiry, we investigated human neonatal lung tissue. We observed that the lungs of human newborns exposed to intrauterine infection had significantly higher numbers of PD1^+^ cells compared to specimens who died from non-infectious causes. Together, these data suggest that PD1/PDL1, a pathway typically thought to govern adaptive immune processes in adult animals, can modulate the largely innate neonatal pulmonary immune response to experimental septic insult. The potential future significance of this area of study includes that PD1/PDL1 checkpoint proteins may be viable therapeutic targets in the septic neonate.

## Introduction

Sepsis remains a devastating illness with high morbidity and mortality despite diagnostic and supportive therapeutic advances over the past several decades (1, 2). The costs of sepsis on the healthcare system remain economically burdensome (3). Among surgical patients, an intra-abdominal source of the sepsis is a predominant driver of this mortality. These effects are most profoundly noted in the extremes of age—namely the geriatric and neonatal cohorts (4, 5). For the neonatal cohort specifically, mortality has remained relatively unchanged despite significant advances in neonatal critical care (6, 7). Compared to the adult cohort, there is a relative paucity of data regarding the neonatal response to an intra-abdominal septic challenge, let alone an age-specific definition of sepsis criteria (8). The detrimental effects of neonatal sepsis are thought to arise from an inadequate response of the immature immune system (9).

Sepsis induces an overwhelming cellular and cytokine cascade that often disrupts the steady-state, or balance, of the immune system (10). When this dysfunction occurs, the result often includes end-organ damage (11). Not only is remote septic organ failure attributed to the bacterial burden of the septic source itself, it is also attributed, perhaps more predominantly so, to multi-factorial immune-cell disharmony (12). Impaired barrier function follows, and circulating neutrophils influx into and habitate within those affected remote organ systems (13).

As an example of this septic organ dysfunction, indirect acute lung injury has been shown to correlate with direct insult to pulmonary endothelium (14). The damage to the microvasculature of the lungs is attributed to this interaction between the endothelial cells and infiltrating neutrophils, and the cell injury/death that follows (15). This indirect acute lung injury contributes to morbidity and mortality in both adult patients and in the murine double-hit model of sepsis and hemorrhage (16, 17).

Several models have been described and tested in an attempt to replicate the septic picture found in adults. Cecal Ligation and Puncture (CLP) alone and in combination with hemorrhage for a double-hit scenario have been used predominantly in the Ayala lab (18). The intention of CLP alone is to mimic a clinical picture of a polymicrobial intra-abdominal sepsis, such as perforated diverticulitis (19). The murine models used to mimic adult sepsis are not seamlessly translated to the neonatal model, however.

Although the causes of intra-abdominal sepsis in children are numerous, there are a relatively limited number of etiologies in the newborn population (20). The numerous contributing factors have been distilled down to four pillars: (1) hypoxia and ischemia, (2) bacteria, (3) hypothermia, and (4) formula feeding (21). In this respect, Cecal Slurry (CS) is the purposefully designed reductionist approach to intra-abdominal sepsis. It can be applied across most forms of abdominal sepsis pathologies because it focuses primarily on the pure aspect of the polymicrobial/bacterial burden in the peritoneum and the septic sequelae (22). It was first applied to the neonatal murine model by the Wynn/Moldawer labs and has gained traction as a validated model for studying the immunology of neonatal sepsis (23).

There is an almost overwhelming volume of protein contributors to the inflammatory cascade in sepsis. It is recognized that several key regulators of the immune system, often small cell-surface receptors, exist. Programmed cell death receptor-1 (PD1) has arisen as such a key component in both human and animal models. It is a checkpoint protein first discovered by Ishida et al. at Kyoto University, and is known to be involved in both pro- & anti-inflammatory cascades (24, 25). Clinically, human serum samples had increased PD1 expression in adult septic shock (26). Conversely, adult patients who went on to survive Acute Lung Injury exhibited lower levels of PD1 (17).

We have previously established that, among adult mice, PD1 or PDl ligand 1 (PDL1) gene deletion confers a survival advantage following sepsis (27). More recently, we have demonstrated the improved survival effect of PD1 gene deletion on neonatal pups following septic challenge–the neonatal population being a distinct cohort from an immunological perspective, given their under-developed adaptive immune system (28). This led us to the question: what is contributing to the improved survival witnessed in PD1 deficiency among murine neonates after sepsis? On gross necropsy of these mice, the lungs are markedly more edematous and hemorrhagic in the septic cohorts among wildtypes. This injury was less pronounced among the PD1 knock out mice.

Based on these background data, we hypothesize that PD1 plays an integral role in the immune-mediated lung damage induced by CS, and modulation of this checkpoint protein should improve survival after septic challenge. We suggest that improved sepsis survival seen in PD1^−/−^ pups is driven by end-organ effects, specifically sepsis mediated changes in the lung. Further, building on the understanding that innate immunity is emphasized at the neonatal developmental stage, we contend that PD1 induces neutrophilic burden in the lung, which regulates expression of its ligand PDL1 and contributes to compromised parenchymal integrity.

## Materials and Methods

### Animals

Wild type (WT) C57BL/6J mouse pups were bred from adult mice (Jackson Laboratory, Bar Harbor, ME). Wild type mice deficient in PD1 (PD1^−/−^) were used to breed the knock-out pups (kindly provided by Tasuku Honjo, Kyoto University, Kyoto, Japan, through Megan Sykes at Massachusetts General Hospital, Boston, MA). Mice were bred at the Rhode Island Hospital (RIH) rodent facility and received standard care and diet *ad libitum*. Neonatal pups between 5 and 7 days old were utilized for each experiment in accordance with the Institutional Animal Care and Use Committee of Rhode Island Hospital (AWC# 5064-18), the Animal Welfare Act, and National Institutes of Health guidelines for animal care and use.

### Animal Model of Sepsis

We employed the cecal slurry (CS) model for murine neonatal sepsis based on the previously described model from the Moldawer lab, and adapted for utilization in our experiments (23, 28). In brief, an adult male WT mouse served as the donor of cecal contents for each experiment. Contents were combined in a weight-based manner with 5% dextrose to solution in order to create the cecal slurry of 80 mg/mL concentration. Pups received an intra-peritoneal injection of this CS (1.3 mg/g body weight to produce LD_70_) or a Sham (Sh) injection of equivalently weight-based crystalloid solution. For further details, please refer to Young et al. (28).

### Survival Study

A 7-day survival study was undertaken to compare mortality as the primary outcome. WT, PD1^−/−^, and PDL1^−/−^ pups underwent either Sh or CS injection, followed by a period of observation. Pups were examined approximately every 6 h over the initial 48-h period, followed by observation approximately every 12 h for the subsequent 5 days.

### Wet: Dry Weights

Twenty-four hours after Sh or CS, whole lungs were harvested and weighed. This immediate weight was documented as the wet weight. Serial dry weights were measured starting at 24 h following harvest and were concluded once a difference between serial weights was no longer detected. The ratio of the Wet:Dry weights was then calculated.

### Bacterial Culture

As per the animal model, pups were injected with either CS or Sh crystalloid, and observed for 24 h. Following this period, lung tissue was homogenized and 100-μl samples were plated onto sheep's blood agar plates. After 24 h of incubation at 37.5°C, the plates were assessed and colony forming units (CFUs) were quantified.

### Histology

Lungs were harvested at 24 h following injection (either Sh injection or CS) and maintained in formalin for sectioning. The RIH Core Research Laboratory processed these samples into paraffin wax blocks and sectioned them for staining. Sectioned samples were stained with hematoxylin and eosin (H&E) to allow for gross visual comparison of pulmonary architecture.

Additional samples were stained with naphthol esterase to identify granulocytic cells as a marker for neutrophils. Neutrophil presence was assessed by determining the number of neutrophils present per 5 high power fields on these immunohistochemistry slides. All samples were analyzed by an attending pathologist (by MD) blinded to the sample identity.

### Flow Cytometry

The lungs were digested to single cell suspensions using the Miltenyi Biotec lung dissociation kit as per manufacturer's instructions. Cells were counted to establish the absolute numbers of cells within each lung sample from which subpopulation numbers could be calculated. Cell subpopulations were identified by forward and side scatter profile and subsequently gated using monoclonal antibodies (listed below) along with appropriate isotype controls according to both manufacturer's recommendation and our prior publications (27).

The fluorochrome-conjugated monoclonal antibodies used here were: PE-labeled anti-CD3e (clone 145-2C11; T-cells), and APC-labeled anti-Gr1 (clone RB6-8C5; Neutrophils) from eBioscience. In additional experiments, neutrophils were also double stained as being Ly6G^+^ CD11b^+^. PD1 and PDL1 antibodies were utilized to co-stain neutrophils for these checkpoint molecules of interest (AbCam). Samples were processed with our lab's Miltenyi Biotec MACSQuant® flow cytometer. FlowJo (version 9.3.2) was utilized to analyze the data.

### Protein Expression

The expression of the protein platelet endothelial cell adhesion molecule-1 (PECAM-1) and zona occludens-1 (ZO-1) levels were compared in homogenized lung samples from WT and PD1^−/−^ pups following Sh and CS by separating them by polyacrylamide gel electrophoresis, western blot transfer and immunoblot detection as previously outlined in our laboratory (29, 30). Images were digitalized using MultiImage Light Cabinet and the band intensity determined.

### Enzyme-Linked Immunosorbent Assay

Using methods we have previously published (31–33), ELISA was utilized to measure cytokine levels of homogenized lung tissue after 24 h of sepsis. These cytokines include IL-6, IL-10, and TNF-α.

### Endothelial Cell Analysis

After 24 h of sepsis, lungs were harvested and endothelial cells were isolated and cultured. After 3 passages of isolation and culture, endothelial cells were immunofluorescently stained for VE-cadherin, ICAM and ZO-1 as performed previously by the Vascular Research Laboratory, at the Veterans Affairs Medical Center in Providence, RI (34, 35).

### Human Samples

Lung samples from preterm infants exposed to intrauterine inflammation and age-matched controls were obtained from the perinatal autopsy files of Women and Infants Hospital (Providence, RI). The study protocols were approved by the Institutional Review Board and informed consent was obtained in compliance with institutional guidelines (IRBNet # 792344-5). Medical and autopsy records were reviewed for sex, gestational age, weight, placental findings, and cause of death. Infants with congenital, chromosomal, or other anomalies potentially predisposing to pulmonary anomalies were excluded. To study the effects of intrauterine sepsis/inflammation on pulmonary PD/PDL1 expression, infants were divided in 2 groups according to the presence/absence of acute chorioamnionitis (defined as presence of neutrophils within amnion and chorion of extraplacental membranes and chorionic plate) with histopathologic evidence of fetal inflammatory response (i.e., chorionic and/or umbilical acute vasculitis) (Table 1). Formalin-fixed, paraffin-embedded samples were immunostained for PD1 and PDL1 and analyzed by a board-certified perinatal pathologist (by MD) with expertise in neonatal pulmonary pathology who was blinded to the clinical data.

**Table 1:** Infant clinical characteristics and the extent of lung specimen PD1^+^.

**GA at birth (wks)**	**Weight (grams)**	**Sex**	**Still/live birth**	**Duration of life**	**Infection status**	**Cause of death**	**PD1^**+**^/10 (20x) HPF**
23	503	F	L	1 h	No infection	PPROM, extreme prematurity	3
24	703	M	L	11 h	No infection	PA, HMD/RDS, PH	6
23	474	F	L	<1 day (undefined)	No infection	PTL, twin, HMD, PIE, PH	3
24	654	M	L	2 h	No infection	Placental abruption, PPROM, HMD	5
28	1,176	M	S	N/A	No infection	Twin A DiDi pregnancy	2
27	1,242	M	S	N/A	No infection	PA	2
23	496	M	L	4 h	Infection	AFIS	463
23	620	M	L	<1 h	Infection	AFIS	165
26	1,029	F	L	<1 day (undefined)	Infection	AFIS, ACA	13
23	850	F	L	<1 h	Infection	GBS bacteremia, ACA	5
23	573	M	L	9 h	Infection	AFIS, HMD	109
23	550	F	L	<1 h	Infection	AFIS	95
23	490	F	L	7 h	Infection	GBS bacteremia, ACA	688
23	705	M	L	5 h	Infection	AFIS, neutrophils airspaces	303
23	608	M	L	3 days	No Infection	Extreme prematurity, HMD	2
26	1,116	F	L	2 days	No Infection	TTTS recipient, IMN, HMD	5
24	420	M	L	2 days	No Infection	Triplet, early BPD, PH	1
23	472	F	L	3 days	No Infection	HMD, PIE, twin	5
23	680	F	L	2 days	No Infection	PH	24
27	594	M	L	4 days	No Infection	CHF, pulmonary hypoplasia, placental massive PVFD	2
24	608	F	L	10 days	Infection	Staph sepsis, PNA, ACA, DiDi twin	7
27	614	M	L	7 days	Infection	Klebsiella sepsis, PNA, IUGR	55
25	854	M	L	18 days	Infection	E. coli sepsis, NEC, chronic PA, IUGR	10
27	1,156	M	L	8 days	Infection	E coli sepsis, meningitis, BPD	7
25	910	M	L	13 days	Infection	E. coli sepsis, PA	364
24	370	M	L	2 days	Infection	Sepsis, IVH, PA, PH, HMD, PIE, IUGR	3
24	751	F	L	16 days	Infection	Pseudomonas sepsis, PNA	98
24	931	M	L	15 days	Infection	PNA, staph sepsis, severe ACA, early BPD	24
23	449	F	L	4 days	Infection	Staph sepsis, Didi twin, massive PH	6
23	495	M	L	8 days	Infection	Staph sepsis, intestinal perforation, PH, DiDi twin	14
24	579	M	L	9 days	Infection	Pseudomonas aeruginosa sepsis, IUGR	10
24	992	F	L	4 weeks	Infection	NEC, early BPD, staph & candida sepsis	2
25	475	F	L	6 days	Infection	ACA, staph sepsis, IUGR	41
23	530	F	L	7 days	Infection	NEC, staph sepsis, DiDi twin, bilateral PNA	52
25	809	M	L	8 days	Infection	NEC, PNA, HMD	64

*ACA, Acute Chorioamnionitis; AFIS, Amniotic Fluid Infection Syndrome; BPD, Bronchopulmonary Dysplasia; CHF, Congestive Heart Failure; DiDi, Dichorionic Diamniotic; GA, Gestational Age; GBS, Group B Strep; HMD, Hyaline Membrane Disease; HPF, High Power Field; IMN, Ischemic Myocardial Necrosis; IUGR, Intrauterine Growth Retardation; IVH, Intraventricular Hemorrhage; NEC, Necrotizing Enterocolitis; PA, Placental Abruption; PH, Pulmonary Hemorrhage; PIE, Pulmonary Interstitial Emphysema; PNA, Pneumonia; PROM, Preterm Premature Rupture of Membranes; PTL, Premature Labor; PVFD, Perivillous Fibrin Deposition; RDS, Respiratory Distress Syndrome; TTTS, Twin to Twin Transfusion Syndrome*.

Here we specifically stained for PD1 on post-mortem lung tissue of neonates who died either immediately after birth, i.e., within 24 h, or in a delayed manner, i.e., after 48 h of life. Presence of infection as delineated by diagnosis of chorioamnionitis, bacteremia, pneumonia, NEC, or amniotic fluid infection syndrome (AFIS). Other non-infectious causes of death included myocardial necrosis, pulmonary hemorrhage, placental abruption, and hyaline membrane disease with associated respiratory distress syndrome (HMD/RDS). Finally, in an effort to quantify the cumulative change in the numerical density of PD1 positive (PD1^+^) cells among groups the number of PD1^+^ cells per 10 high powered fields (HPF), at 20× magnification, was determined for each lung autopsy specimen.

### Statistical Analysis

Statistical analyses were accomplished with SigmaPlot 12.5 (Systat Software, San Jose, CA). Categorical data was assessed using χ^2^ or Fisher's exact test. The data were tested for normality and normally distributed data sets were analyzed by *T*-test; non-normally distributed data sets were analyzed by Mann-Whitney U in order to assess continuous data across 2 groups. Continuous data are expressed as mean and standard error of the mean. One-way analysis of variance (ANOVA) with Holm–Sidak *post-hoc* analysis was used for continuous data across multiple groups. Survival analysis was undertaken using Kaplan Meier curves. Alpha was set to 0.05.

## Results

### Survival Study

**Figure 1:**
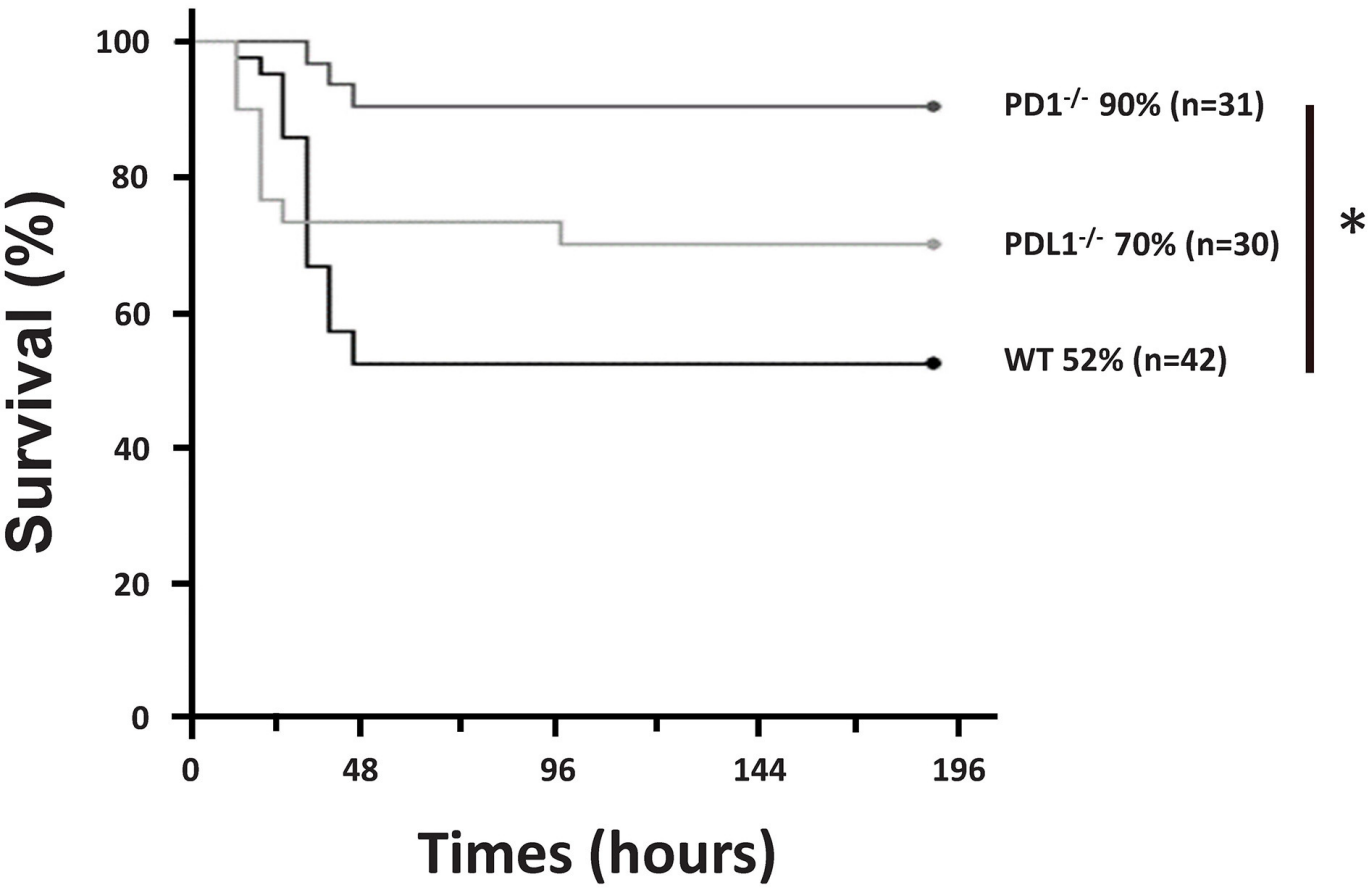
PD1 deficiency improved survival after cecal slurry (CS). C57BL/6 WT and PD1 and PDL1 deficient mice were subjected to CS, and survival was recorded. PD1^−/−^ mice had a 90% survival rate compared to WT counterparts who only had 52% survival over a 7-day survival study. Those with deletion of PDL1 had a survival of 70% which was not significantly improved from the WT group. (*P < 0.05 vs. WT, log-rank survival analysis; *n* = 31–42/group).

In agreement with our recent publication (28), we found that, following CS in WT pups, survival was noted to be 52% by 7 days (*n* = 42). However, in the PD1^−/−^ pups, survival was noted to be markedly improved to 90% (*p* = 0.001) following septic challenge (*n* = 31). Similarly, survival analysis (*n* = 30) of PDL1^−/−^ neonatal mice after CS resulted in 70% survival. While this, like the PD1^−/−^ group, trended toward improved survival over the WT group it was not statistically different (Figure 1). Inasmuch, the remainder of the study focused on the impact of PD1, not PDL1 gene deficiencies impacts here.

### Lung Edema

**Figure 2:**
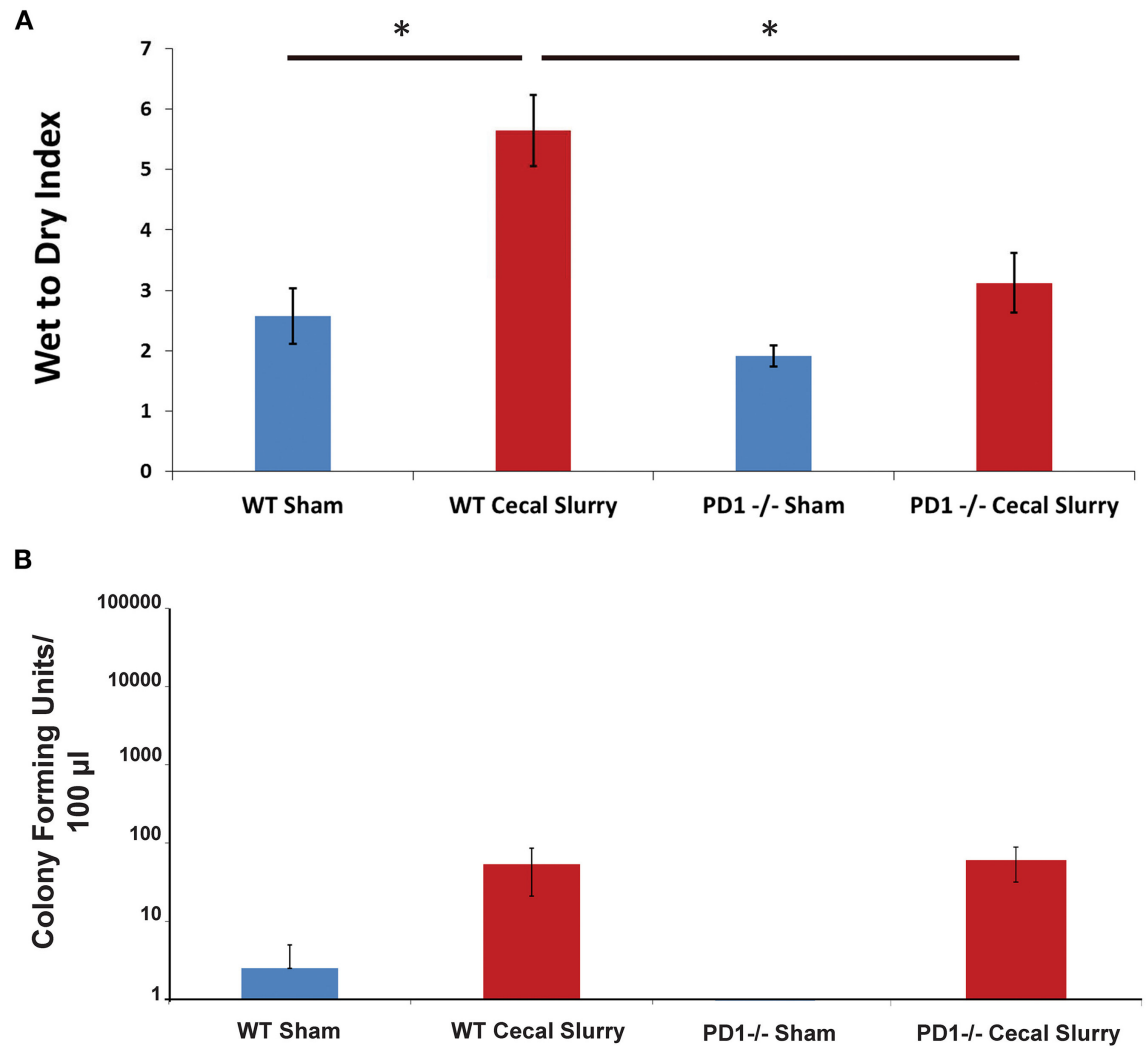
Pulmonary edema as measured by Wet to Dry index of lung weights **(A)**. Lungs of wildtype (WT) mice following cecal slurry (CS) had significantly more edema compared to lungs of the PD1^−/−^ mice after CS. Mean ± SEM, *n* = 5–13 per group. (*P < 0.05 vs. WT CS group; *t*-test). Bacterial burden in the peritoneal cavity **(B)** among wildtype (WT) mice. The sham group (*n* = 4) and the cecal slurry (CS) group (*n* = 18) had no significant difference in Colony Forming Units (CFU) measured after culture of peritoneal lavage. Bacterial burden among PD1^−/−^ pups after sham (*n* = 7) or CS (*n* = 18). Similar to the WTs, there was no significant difference in peritoneal lavage bacterial CFU counts after sham or CS injections. Mean ± SEM; *P* > 0.05 (not different) vs. sham group (PD1^−/−^ CS); *t*-test.

Among WT pups, lung edema (as measured by Wet:Dry weight ratio/index) was noted to increase after CS compared to Sh (*p* < 0.05). However, among the PD1^−/−^ pups, there was no difference in lung edema between Sh and CS groups. In addition, PD1^−/−^ CS pups showed significantly less edema compared to WT CS groups (*p* < 0.05; Figure 2A).

### Lung Bacterial Burden

Interestingly, unlike our previously reported rise in peritoneal bacterial burden (28), there was minimal bacteria detected in the homogenized lung samples across all 4 groups, Sh and CS in both WT vs. PD1^−/−^ pups (Figure 2B).

### Lung Histology

**Figure 3:**
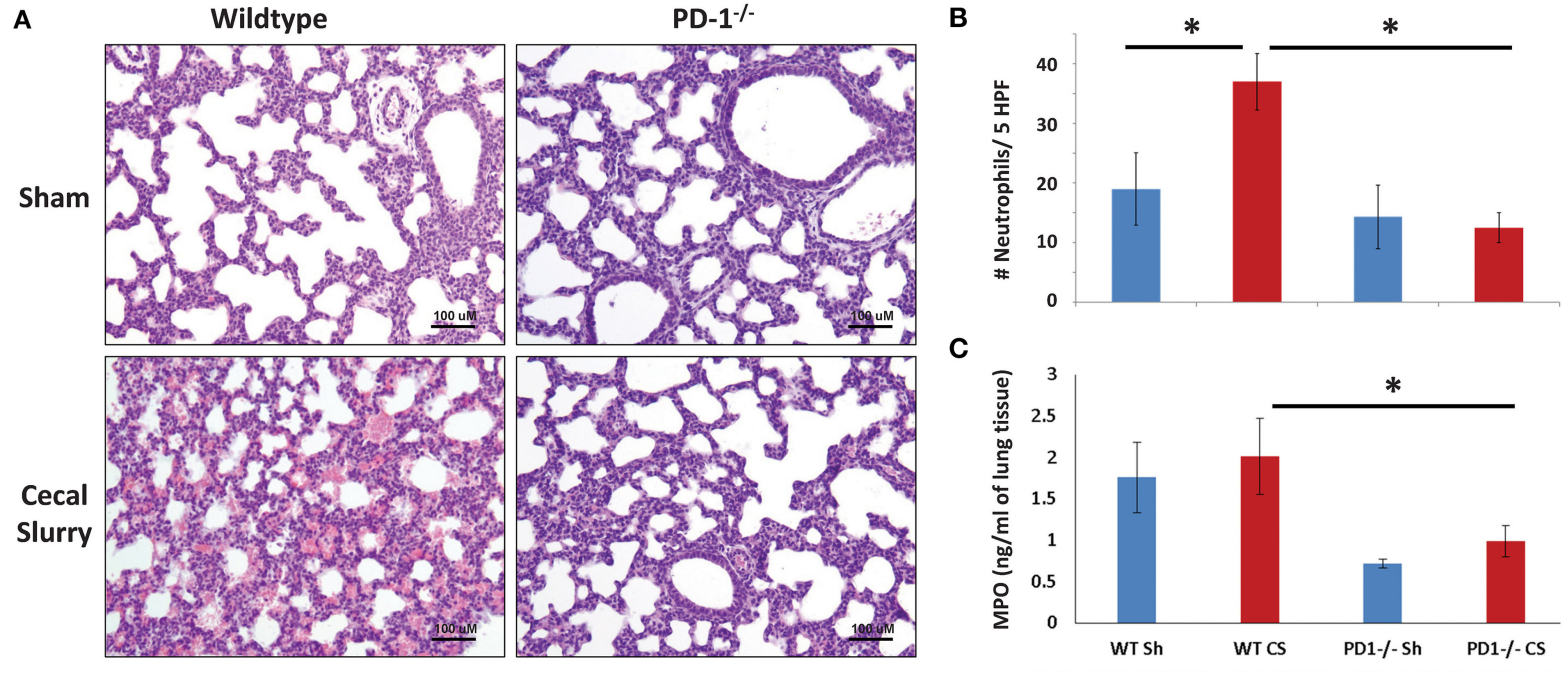
Histology of lung parenchyma, representative image of each category (Original magnification: 200×; scale bar: 100 μm) **(A)**. On qualitative analysis of samples, alveolar thickening appears attenuated in the cecal slurry (CS) group among PD1^−/−^ mouse lungs compared to wildtypes (WT). Neutrophil quantification based on naphthol esterase staining **(B)**. Unlike the WT, where a significant influx of neutrophils to the lung after CS, no significant change in neutrophil count after CS among the PD1^−/−^ population. While there was a trend toward an increase in lung tissue Myeloperoxidase (MPO) levels when compared to WT sham (WT Sh), this did not reach statistical significance **(C)**. However, both PD1^−/−^ Sh and CS groups were both markedly lower than the WT Sh and CS group, respectively. Though, again, not different from each other. Mean ± SEM, *n* = 5–8 per group in **(B)** and *n* = 10–16 in **(C)**; *P < 0.05 vs. WT CS group; ANOVA with Holm–Sidak *post-hoc* analysis.

No gross architectural changes or diffuse alveolar damage were noted following CS in either the WT or the PD1^−/−^ pups. However, CS did induce mild morphological changes in the lungs of the WT pups. This was most markedly noted in alveolar thickening and mildly increased intra-alveolar hemorrhage. These alterations were not noted in the lungs of PD1^−/−^ pups after CS (Figure 3A).

Naphthol esterase staining revealed an increased presence of neutrophils in the lungs after CS among the WT pups. Unlike the WTs, there is no significant change in neutrophil influx between Sh and CS groups among the PD1^−/−^ population (Figure 3B).

As a functional assessment, beyond a quantitative determination of neutrophil presence in the lungs, we undertook to evaluate MPO expression in the lung. This is an indirect measure of neutrophilic metabolism. Levels were lowest at 4 h after septic challenge (or Sh), with an increase at 12 h and highest levels at 24 h across all groups (Figure 3C; data for 4 & 12 h not shown). Although there was a trend of increase after sepsis in both mice strains, with attenuation among PD1^−/−^ mice, this finding did not reach statistical significance.

### CD18, CD177, and PECAM-1 Expression on Pulmonary Neutrophils

**Figure 4:**
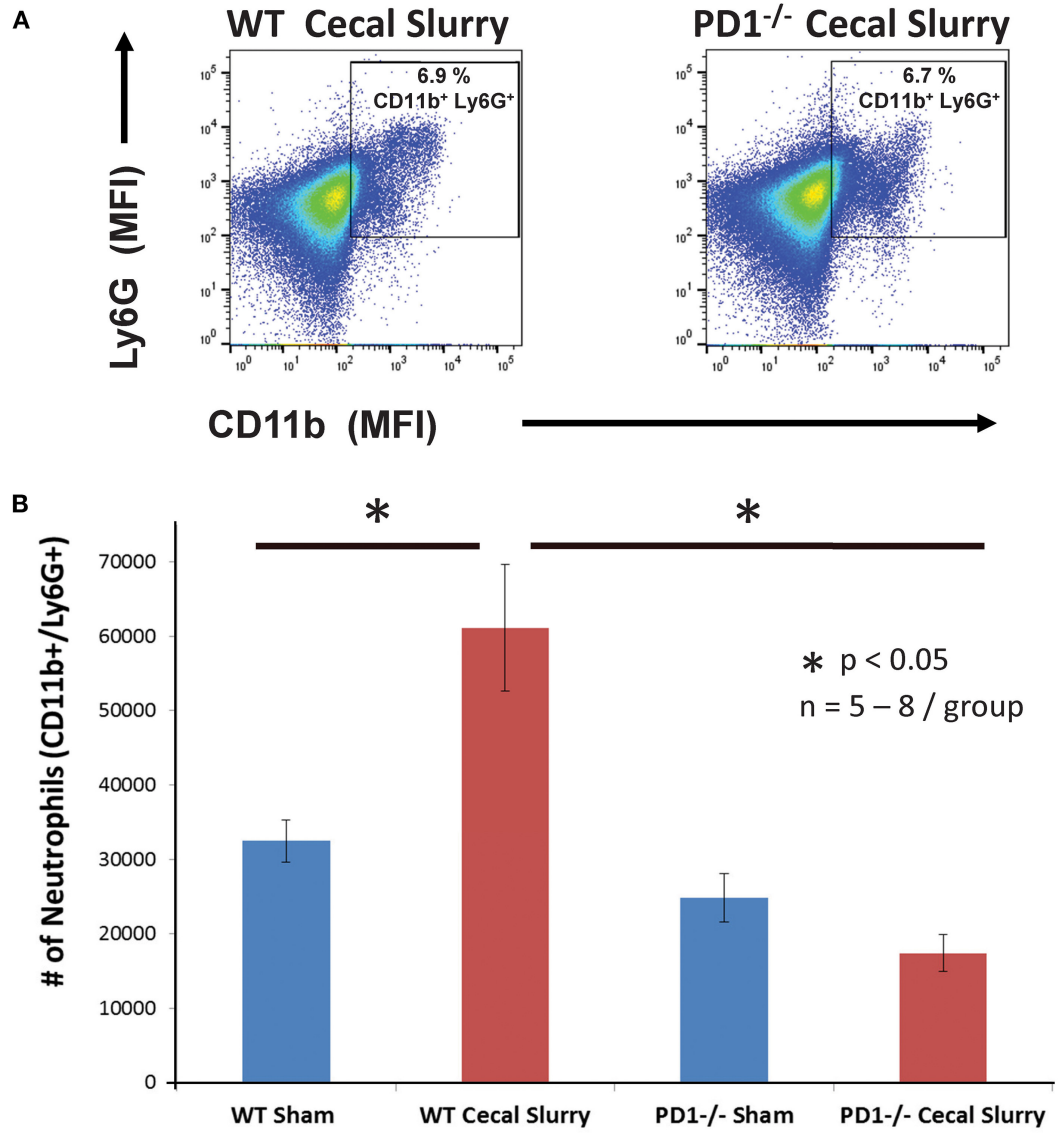
Neutrophils increase in wildtype (WT) but not PD1^−/−^ cecal slurry (CS) mouse lung tissue. **(A)** Flow cytometry with neutrophils being gated as CD11b and Ly6G-positive among lung samples for WT-CS group vs. PD1^−/−^ CS mouse (paired representative dot-plots). Mean Fluorescence Intensity (MFI). The summary data from repeated experiments indicate a marked rise in the neutrophil number **(B)** in WT CS lung tissue when compared to Shams. However, this was not changed between PD1^−/−^ Sh and CS animals. Mean ± SEM, *n* = 5–8 per group; *P < 0.05 vs. WT CS group; ANOVA with Holm–Sidak *post-hoc* analysis.

On flow cytometric analysis, we gated for pulmonary neutrophils as CD11b^+^Ly6G^+^ (Figure 4A). There was an increased neutrophil count after CS among WT pups, but no difference among the PD1^−/−^ pups (Figure 4B).

**Figure 5:**
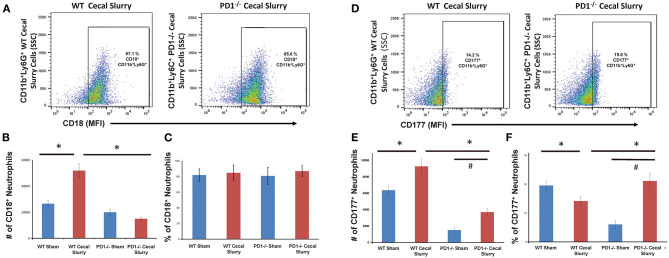
Flow cytometry with CD18^+^ neutrophils being gated as CD11b and Ly6G-positive among lung samples **(A)** for wildtype (WT)-cecal slurry (CS) group vs. PD1^−/−^ CS mouse (paired representative dot-plots). The absolute number of CD18^+^ cells **(B)** was significantly higher among WT-CS mouse lungs compared to the other groups. However, on analysis of the percentage of CD18-positive cells **(C)**, there was no difference among the groups. Flow cytometry with CD177^+^ neutrophils being gated as CD11b and Ly6G-positive among lung samples **(D)** for wildtype (WT)-cecal slurry (CS) group vs. PD1^−/−^ CS mouse (paired representative dot-plots). The absolute number of CD177^+^ cells **(E)** was highest among WT-CS mouse lungs, but this rise was mitigated in the PD1^−/−^ CS group. Analysis of the percentage of CD177^+^ cells **(F)**, showed differences between WT Sh and WT CS as well as PD1^−/−^ Sh and PD1^−/−^ CS groups. Mean ± SEM, *n* = 5–8 per group; *P < 0.05 vs. WT CS group; #*P* < 0.05 vs. PD1^−/−^ CS group; ANOVA with Holm-Sidak *post-hoc* analysis.

We next gated for CD18 expression upon these pulmonary CD11b^+^Ly6G^+^ neutrophils (Figure 5A). Although there was no difference in the percentage of CD11b^+^Ly6G^+^CD18^+^ cells among the groups, there was an increased number of CD18^+^ neutrophils in WT mouse lung cells following CS compared to the other three (WT Sh, PD1^−/−^ Sh, and CS) groups (Figure 5B). However, on analysis of the *percentage* of CD18^+^ cells, there is no difference among the groups (Figure 5C).

We next assessed CD177 expression upon pulmonary neutrophils (Figure 5D). In WT CS pups, fewer pulmonary neutrophils were noted to express CD177 compared to Sh group. However, in PD1^−/−^ CS pups, the reverse was observed, wherein a considerably greater percentage of pulmonary neutrophils expressed CD177 (Figure 5E). When accounting for cell numbers, this corresponded to an increase in total number of CD177^+^ neutrophils in lungs following CS compared with Sh for both WT and PD1^−/−^ pups (Figure 5F).

**Figure 6:**
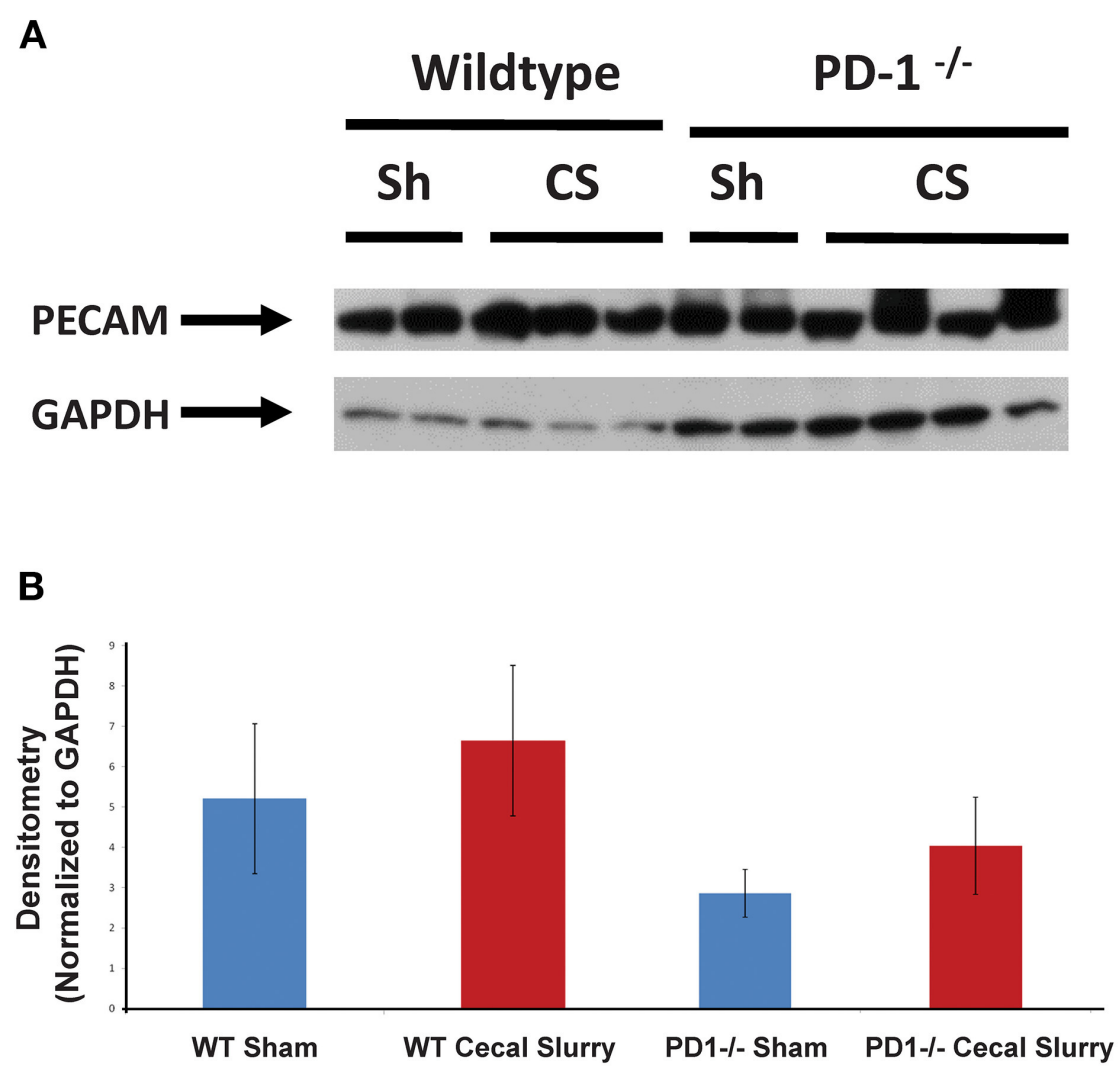
PECAM-1 expression in the lung. Western blot analysis (a representative blot provided) **(A)** showed no significant difference among the groups in terms of PECAM-1 expression [**(B)**, provide summary data of repeat assessments of PECAM-1 expression as a ratioed to the housekeeping gene GAPDHs' expression]. Mean ± SEM, *n* = 6–9 per group; *P* >0.05 vs. Sham group; ANOVA with Holm–Sidak *post-hoc* analysis.

No difference was noted in PECAM-1 expression across WT or PD1^−/−^ pups following Sham or CS injections (Figures 6A,B). In a previous study, PECAM-1 levels in human neonatal lung tissue were significantly increased following intubation, suggesting endothelial cell proliferation in response to stress of mechanical ventilatory methods (36).

### Lung Zona Occludens-1 Expression

**Figure 7:**
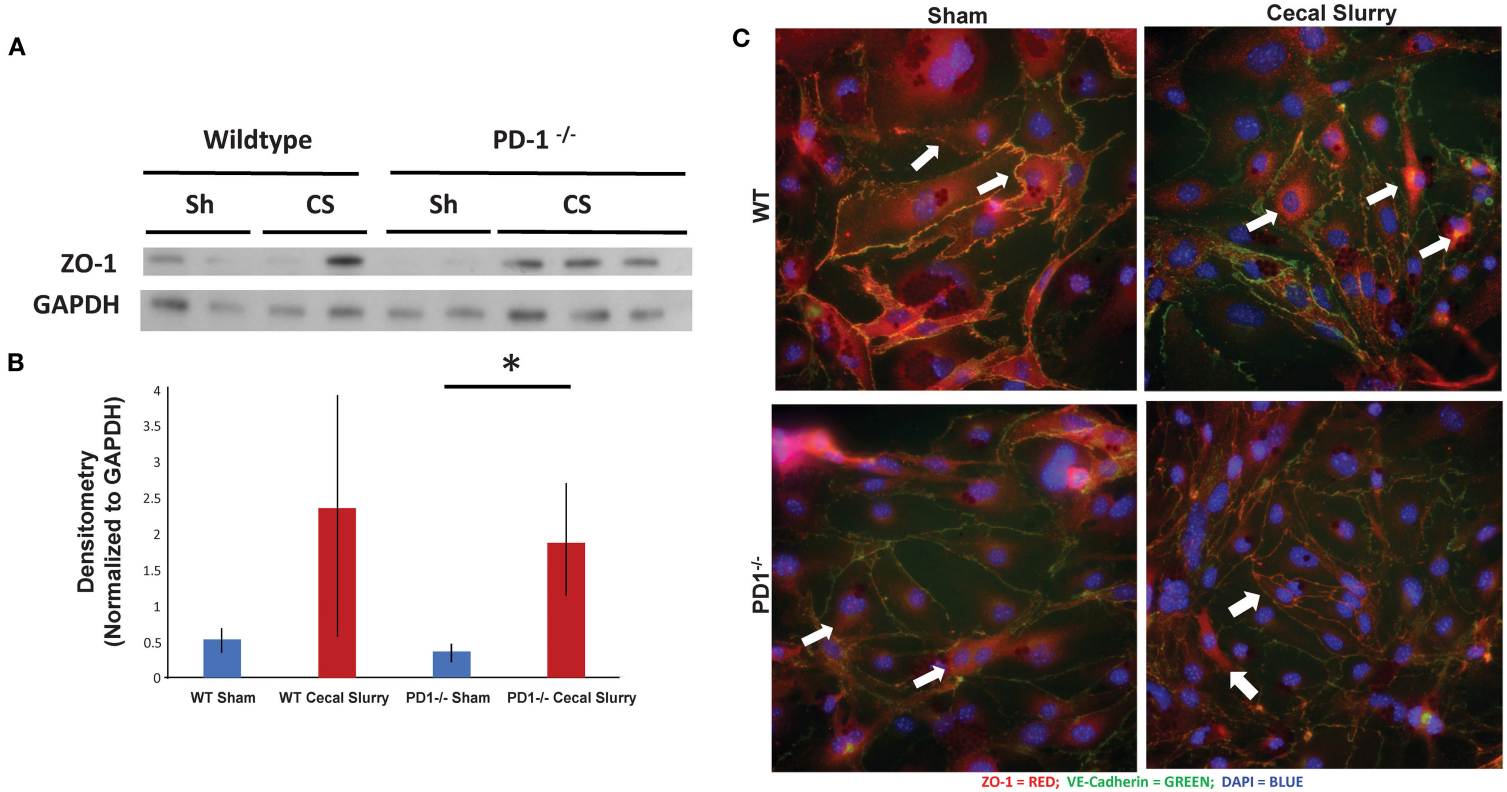
ZO-1 expression in the lung. Western blot analysis (a representative blot provided) **(A)** while showing a trend toward increased ZO-1 expression, relative to GAPDH, in the lungs of the wildtype (WT) cecal slurry (CS) treated mice, it was not a statistically significant difference from the WT sham (Sh) animal group. Further, while ZO-1 expression in PD1^−/−^ CS lung tissue was markedly increased over the PD1^−/−^ Sh group, this rise was not different from the WT CS group's ZO-1 levels [**(B)**, provide summary data of repeat assessments of ZO-1 expression). Mean ± SEM, *n* = 3 per group; *P < 0.05 vs. PD1^−/−^ CS group; ANOVA with Holm–Sidak *post-hoc* analysis. Representative images showed that ZO-1 undergoes peri-nuclear consolidation on endothelial cell monolayers obtained from the lungs of neonatal mice exposed to 24 h of CS when compared to Sh treatment among wildtype (WT) animals **(C)**. Alternatively, the ZO-1 staining maintains its more peripheral location seen in endothelial cell monolayers derived from WT Sh mice irrespective if they are isolated from PD1^−/−^ Sh or CS treated animals.

ZO-1 expression in the lung, based on homogenized lung tissue 24 h post-injection, was increased after sepsis in both WT and PD1^−/−^ mice, however, this increase was not statistically significant (Figures 7A,B). This was counter to our hypothesis based on prior observations in adult mice subjected to an experimental model of indirect acute lung injury that endothelial cell compromise after sepsis would be mediated by downregulation of ZO-1 (37, 38).

### Endothelial Cell Culture

To explore the changes in adhesion molecules in a more detailed fashion, we immunofluorescently stained ZO-1 in isolated and cultured endothelial cells from lungs after 24 h of Sh or CS injection (Figure 7C). Of note, the distribution of ZO-1 was altered after sepsis, but this change is muted among the PD1^−/−^ murine lungs.

### Lung Tissue Cytokine Expression

**Figure 8:**
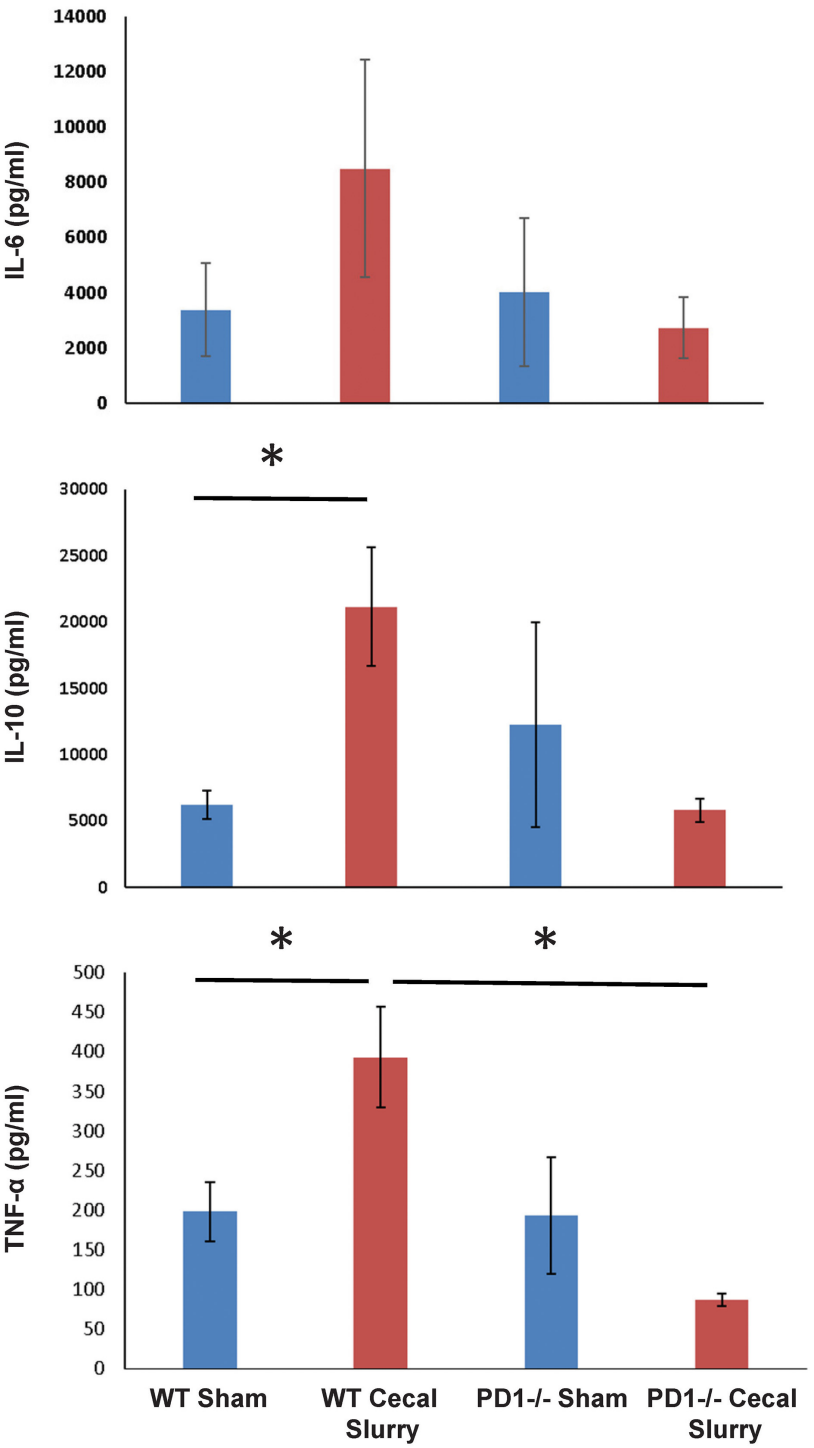
The absence of PD1 gene expression suppresses expression of cytokines IL-10 and TNF-α after CS compared to WT CS. A similar pattern is witnessed in IL-6 among PD1^−/−^ mice after CS but did not reach significance. Mean ± SEM, *n* = 6 per group; *P < 0.05 vs. wildtype (WT) CS group; *t*-test.

We next measured cytokine expression in homogenized lung tissue after 24 h of Sh or CS mice to test whether the inflammatory micro-environment would be altered in the presence or absence of PD1. Sepsis elevated IL-10 and TNF-α among WT mice, but this effect was lost among PD1^−/−^ murine lungs. Although a similar pattern was found for IL-6, this did not reach statistical significance (Figure 8). A limitation of the animal study design is that tissue of survivors (but not those mice who perished) were analyzed; with this being a neonatal model our numbers were limited by how many mice were born within each litter, each litter being analyzed separately (i.e., samples were not batched across multiple litters). For cytokine analysis, due to variance of results, this setup led to an underpowered result.

### Human Neonatal Lung Pathology Analysis

**Figure 9:**
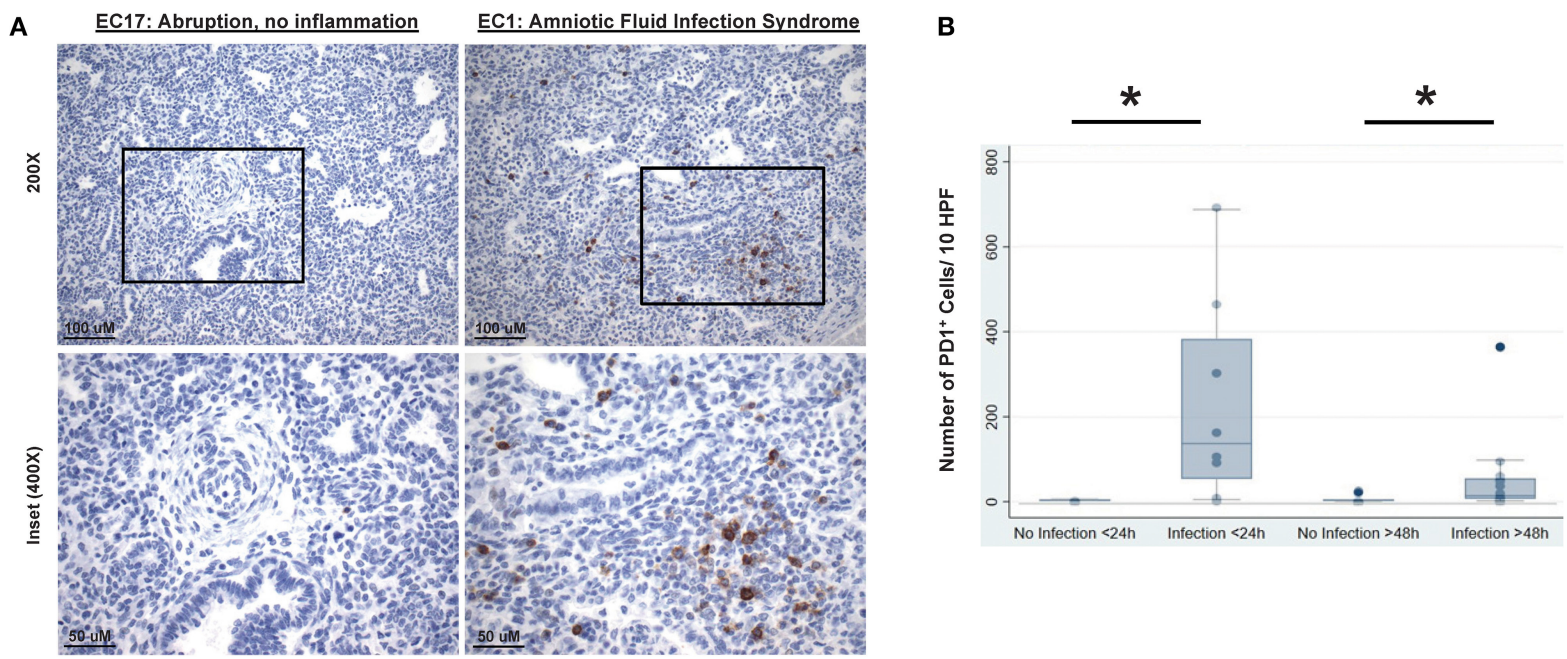
Representative lung autopsy specimens derived of a non-septic (patient EC17: cause of death “abruption, no inflammation”) and septic newborn (patient EC1: cause of death “amniotic fluid infection syndrome”) who died either immediately after birth, i.e., within 24 h, stained for their expression of PD1 **(A)**. Original [top half **(A)**] image magnification ×200, scale bar: 100 μm; inset [lower half **(A)**] at magnification ×400, scale bar: 50 μm. The cumulative change in the numerical density of PD1 positive cells per 10 high powered fields (HPF) **(B)**, at 20× magnification, is presented for lung autopsy specimens from either non-septic (No Infection) or septic (Infection) newborn whom died either immediately after birth, i.e., within 24 h (<24 h), or in a delayed time, i.e., after 48 h (>48 h) of life. Data is provided as box and whisker plot; *P < 0.05, “No Infection <24 h (*n* = 6)” vs. “Infection <24 h (*n* = 8)” group or “No Infection >48 h (*n* = 7)” vs. “Infection >48 h (*n* = 15)” group; Kruskal–Wallis test.

In order to assess the clinical relevance of these observations made in rodents, PD1/PDL1 expression was studied in lung specimens of newborn infants with or without evidence of intrauterine inflammation/sepsis who succumbed within 24 h of birth. Previous human study on neonatal lungs found that wet weights or edema is associated with pathologically significant inflammation of lung parenchyma (39). Here we found that lungs of infants with evidence of antenatal inflammation/infection [*N* = 8; “Duration of Life” group = Infection <24 h (Table 1)] uniformly displayed abundant PD1-immunoreactive cells, often preferentially located in peribronchial location or in lymphoid aggregates in interstitium and interlobular septa (Figure 9A, “EC1: Amniotic Fluid Infection Syndrome”). In contrast, the lungs of age-matched control infants without evidence of antenatal inflammation/no infection [*N* = 6; “Duration of Life” group = No Infection <24 h (Table 1)] were virtually devoid of PD1-immunoreactive cells (Figure 9A, “EC17: Abruption, no inflammation”). The numerical density of PD1-positive cells as summary data is depicted in Figure 9B. In this respect, newborns dying within 24 h of birth, diagnosed as infected/septic had a pronounced increase in PD1 positive staining (Figure 9B). Further, while reduced in those newborns that survived beyond the first 24 h, a PD1 positive signal was still evident even in the later mortalities if they too were diagnosed with infection/sepsis.

## Discussion

Lung injury in clinical sepsis is a common and often devastating end-organ injury following intra-abdominal polymicrobial insult. The neonatal sepsis model of CS induces gross changes in the lung upon necropsy. To better assess this injury beyond subjective direct visualization, we undertook to quantify the edema in the lungs as a rudimentary marker of injury. Edema is clearly evidenced in the lungs of septic WT mice, however, among the PD1^−/−^ group, the statistically significant increase that we witnessed in the WT is now lost. This suggests that deletion of the PD1 gene has a protective effect against pulmonary fluid accumulation after remote septic insult.

This supports what we have seen in human septic lung injury. Blood leukocytes of adult septic patients expressed higher levels of PD1 than their non-septic counterparts. Septic patients who survived a component of indirect Acute Lung Injury had lower levels of PD1 expression compared to non-survivors (17). Our question was: do newborns with sepsis also incur changes to the lung that correspond to PD1:PDL1 expression?

Having established a clear role for PD1 in attenuating neonatal lung injury in a septic mouse model, we next endeavored to correlate this with human lung samples and to establish clinical relevancy for the PD1 and PDL1 interaction in the neonatal septic state. With IRB approval, we forged a clinical collaboration that allowed us to analyze autopsy samples of septic and non-septic newborns that had been previously biobanked, and to stain their lung tissue for our checkpoint molecule of interest. We analyzed a total of 20 randomly selected human lung specimens, with non-septic lungs consistently expressing less PD1, and with a less consistent attenuated expression of PDL1. This human data led us to ask: what can experimental sepsis tell us about the underlying mechanism of remote lung injury in neonates?

On histology, the WT mice demonstrated clear lung injury after CS sepsis. In the PD1^−/−^ mice compared to WT, however, the alveolar thickening appears mildly attenuated. To characterize the histology further, we quantified the neutrophils present in the lungs by utilizing naphthol esterase immunohistochemistry. We saw that CS is associated with increased neutrophil influx, suggesting that this one-hit model of intra-abdominal sepsis was inducing an indirect effect of increased neutrophil presence in the pulmonary parenchyma. Unlike the WTs, there is no significant change in neutrophil influx after CS among the PD1^−/−^ population. This reinforced our supposition that PD1 gene deletion is protective against the lung injury incurred after CS, with neutrophil influx being a marker of this damage.

What mechanisms promote increased cellular congestion in the lung parenchyma? We hypothesized that adhesion molecule(s), like Zona occludens-1 (ZO-1), which promotes inter-cellular integrity (40, 41), may be compromised in the setting of sepsis and PD1:PDL1 burden in the lung. However, based on protein expression from western blot analysis of homogenized lung tissue, we found that the pattern of amplified ZO-1 expression after sepsis was comparable in both groups, contrary to our hypothesis. This led us to alternatively hypothesize that the differences in lung permeability might not so much be a result of changes in the *level* of ZO-1 expression, but might result from differences in the *pattern* of cellular expression?

To test this hypothesis, we isolated and *ex vivo* cultured neonatal lung endothelial cells from various Sh or CS mice and immunofluorescently stained them for adhesion molecule expression pattern. Among the WT, the ZO-1 staining was noted to be consolidate around the nucleus of the cells derived from septic mice, unlike the comparative Sh animals' cells (where it was mostly at the periphery of the cell surface). In the PD1^−/−^ mouse cells, however, the peripheral location of the adhesion molecule was maintained, even in the setting of CS. We conclude that perhaps the cytokine milieu of the lungs is affecting the way that ZO-1 localizes in cells, either in addition to direct or indirect neutrophilic interaction discussed earlier. In this regard, we noted that IL-6 expression was increased in both groups, but the increase was much less pronounced among the PD1^−/−^ mice. The levels of IL-10 exhibited a similar pattern, as did TNF-α. This suggests a muted inflammatory environment in the setting of PD1 loss.

CD177 was chosen as a neutrophil marker on our flow cytometry studies. Demaret *et al*. previously identified CD177 on human adult peripheral blood neutrophils as being markedly upregulated among septic shock patients compared to healthy volunteer controls. Especially with our neonatal population, the implications of CD177 expression not only on chemotaxis but also on cellular maturation may be a potential area for further exploration (42). CD177 associates with CD18 as part of the neutrophils' Mac-1 β2 integrin. Bai et al. demonstrated that ligation of CD177 downregulated transmigration across vascular endothelium and thus inhibited cellular localization to the source of injury or sepsis (43). Interestingly, this was independent of PECAM-1 expression, suggesting multiple manners in which neutrophil localization may be regulated (43).

With the neonatal reliance on innate immunity to fight bacterial infections, neutrophils should be particularly scrutinized. Pre-term neonates have been shown to be particularly at risk due to impaired neutrophilic migration compared to neutrophils of full-term neonates (44). Raymond et al. have also suggested that not only is chemotaxis impaired among pre-term neonates, but this corresponds to impaired bacterial clearance secondary to diminished pathogen recognition (45). The Moldawer Lab forged significant progress in the model of intra-abdominal neonatal sepsis, one which was expanded upon here to include study of the effects of presence of or genetic deletion of PD1 (46).

PD1 expression on cells may be a distinctive factor in how organ damage and ultimately survival differs in the setting of sepsis. A limitation of our study is that the human data is strictly correlative; we do not have a confirmed mechanism within human neonates to link PD1 expression with lung injury and death. With this stated, our exploration into PD1 expression in human lung tissues opens doors for further more specific investigations into the pathways through which the sequelae of sepsis unfold. Human pre-term neonatal monocytes have been shown on flow cytometry to express increased PD1 during sepsis (47). This amplified PD1 expression correlated with ante-natal steroid administration in this population. For analysis neonates were divided into early-onset and late-onset. Different from our human analysis, late-onset sepsis patient more strongly correlated with PD1 expression compared to early-onset. Further investigation into peripheral vs. pulmonary parenchymal expression of PD1 in infected neonates could provide insight into the immune effects of the associated pathway.

In summary: murine neonatal lungs are sensitive to extra-pulmonary sepsis as an isolated insult. This is in contrast to adult mouse lungs, which require cellular priming to incur evidence of lung injury (48). By deleting PD1 gene expression, we attenuate this lung injury, with less edema, fewer neutrophils, and loss of compensatory ligand upregulation. Both PD1 and PDL1 are highly expressed on neutrophils present in the lung after sepsis, suggesting a direct cellular mechanism for increased cellular pulmonary congestion. And finally, epithelial cell adhesive junction interactions and the lung tissue cytokine microenvironment are disrupted after sepsis, but PD1 deletion reduces these factors closer to a non-septic state.

In previous research, patients were at higher risk for contracting nosocomial infections during their protracted recovery from the initial septic insult (49). This risk correlated with expression of PD1 within their circulation. When PD1 is ligated via binding to one of its 2 ligands, it acts to prevent the activation of immune cells. This causes downregulation of the function of T and pro-B cells, among others. It also contributes to inhibition of macrophage function in the setting of sepsis (25). Conversely, when we somehow inhibit the function of PD1 (either by completely deleting the gene responsible for the protein creation; or by partially blocking it via an antagonizing agent), we can observe these effects. The immune paralysis induced by sepsis and mediated by PD1 is now reversed, and the innate immune system is re-activated to clear or neutralize the septic challenge. Given the emerging central role for PD1 in the septic response in the adult population, we undertook to assess the contribution of this checkpoint protein in the neonatal demographic.

Not only within patients afflicted with infections, but also within patients afflicted with cancer, is the understanding of PD1's relationship with the endothelium important. A study by Li et al. described a flubendazole treatment model of mice afflicted with melanoma and found that it depressed PD1 expression on melanoma cells themselves and also diminished PECAM-1 expression, thus potentially inhibiting angiogenesis amongst cancer cells (50).

Invariant natural killers T cells may provide a cellular mechanism that could explain the results found in our study of the PD1:PDL1 interactions and subsequent cascade. In the same model of murine neonatal sepsis, but among *i*NKT^−/−^ mice, survival was improved following CS. This may have been mediated by an attenuation in end-organ damage including the liver and the site of infectious nidus, namely the peritoneal cavity (51). In contrast, the Wang lab showed that pre-treatment with an *i*NKT cell stimulator actually improved survival and signs of lung injury compared to non-treated wildtypes (52). Inasmuch, further investigation into *i*NKT cells' role in lung injury will be needed to answer the question of whether *i*NKT cells are responsible for the injury patterns, and septic attenuation described above.

With COVID-19 now in our midst, consideration for the future of lung injury across age groups, including even neonatal populations is important. Neonates have tested COVID-19 positive, particularly when born to infected mothers (53). At present we do not know the downstream harm that neonatal COVID-19 infection may inflict on the developing lung. However, here we have demonstrated a possible key to inflammatory processes within neonatal sepsis and lung injury that might be leverage in such individuals. With the goal in mind of unlocking the mechanisms of inflammation/immunosuppression mediating lung injury, we aim to continue this investigation among further human lung samples and with murine cell-line-specific knock-outs. By learning how lung injury in neonates with sepsis is mediated, perhaps therapies targeting PD1 may be included amongst the keychain of answers.

**Figure 10:**
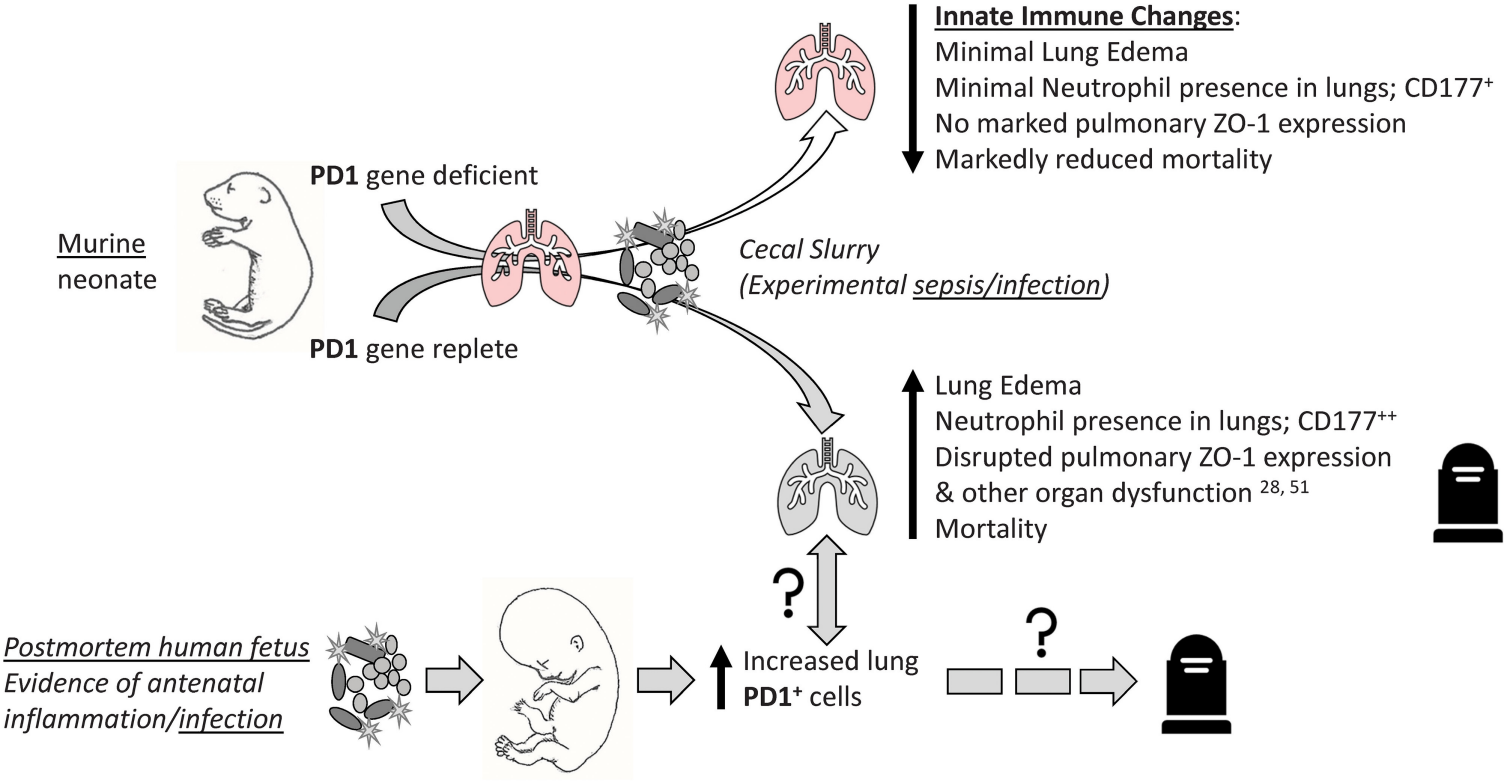
Visual abstract summary of the primary findings of this study. “↑↓” implies general rise/fall in various indices; “?” implies possible association or pathological pathway.

Taken together, we concluded that the PD1:PDL1 ligation compounds lung injury, in part by either directly or indirectly altering aspects of the pulmonary innate immune response, and worsens survival among experimentally septic neonatal mice (Figure 10). This is echoed in part by septic human neonatal histology demonstrating increased pulmonary PD1 levels after sepsis. Together, these data suggest that aberrant signaling through the PD1:PDL1 pathway may be a viable therapeutic target in the septic neonate.

## Data Availability Statement

The original contributions presented in the study are included in the article/supplementary material, further inquiries can be directed to the corresponding author/s.

## Ethics Statement

Human lung autopsy samples were utilized from a specimen bank at Women & Infants Hospital (Providence, RI) of deceased neonates with Institutional Review Board approval (IRBNet # 792344-5). All mouse experiments were performed in accordance with National Institutes of Health guidelines and approved by the Animal Use Committee of Rhode Island Hospital (AWC# 5064-18).

## Author Contributions

EF, C-SC, DH, and AA: design and concept. EF, MD, DH, C-SC, and YC: conduct of experiments. EF, MD, DH, YC, and AA: results analysis and interpretation. EF, C-SC, and AA: manuscript preparation, revisions, and approval. All authors contributed to the article and approved the submitted version.

## Conflict of Interest

The authors declare that the research was conducted in the absence of any commercial or financial relationships that could be construed as a potential conflict of interest.

## Abbreviations

ACA: Acute Chorioamnionitis
AFIS: Amniotic Fluid Infection Syndrome
ANOVA: Analysis of Variance
BPD: Bronchopulmonary Dysplasia
CD: Cluster of Differentiation
CHF: Congestive Heart Failure
CLP: Cecal Ligation and Puncture
CS: Cecal Slurry
DiDi: Dichorionic Diamniotic
GA: Gestational Age
GBS: Group B Strep
HMD: Hyaline Membrane Disease
HPF: High Power Field
IF: Immunofluorescence
IMN: Ischemic Myocardial Necrosis
IUGR: Intrauterine Growth Retardation
IVH: Intraventricular Hemorrhage
NEC: Necrotizing Enterocolitis
PA: Placental Abruption
PD1: Program Cell Death Receptor-1
PDL1: Program Cell Death Receptor-1 ligand
PH: Pulmonary Hemorrhage
PIE: Pulmonary Interstitial Emphysema
PNA: Pneumonia
PROM: Preterm Premature Rupture of Membranes
PTL: Premature Labor
PVFD: Perivillous Fibrin Deposition
RDS: Respiratory Distress Syndrome
Sh: Sham
TTTS: Twin to Twin Transfusion Syndrome
WT: Wild Type.
